# Properties and Applications of Extremozymes from Deep-Sea Extremophilic Microorganisms: A Mini Review

**DOI:** 10.3390/md17120656

**Published:** 2019-11-21

**Authors:** Min Jin, Yingbao Gai, Xun Guo, Yanping Hou, Runying Zeng

**Affiliations:** 1 State Key Laboratory Breeding Base of Marine Genetic Resource, Third Institute of Oceanography, Ministry of Natural Resources, Xiamen 361000, China; jinmin@tio.org.cn (M.J.); Gaiyingbao@tio.org.cn (Y.G.); guoxun0528@163.com (X.G.); Houyanping@tio.org.cn (Y.H.); 2 Southern Marine Science and Engineering Guangdong Laboratory (Zhuhai), Zhuhai 519000, China

**Keywords:** deep sea, extremophilic microorganisms, extremozyme, thermophilic enzyme, psychrophilic enzyme, halophilic enzyme, piezophilic enzyme

## Abstract

The deep sea, which is defined as sea water below a depth of 1000 m, is one of the largest biomes on the Earth, and is recognised as an extreme environment due to its range of challenging physical parameters, such as pressure, salinity, temperature, chemicals and metals (such as hydrogen sulphide, copper and arsenic). For surviving in such extreme conditions, deep-sea extremophilic microorganisms employ a variety of adaptive strategies, such as the production of extremozymes, which exhibit outstanding thermal or cold adaptability, salt tolerance and/or pressure tolerance. Owing to their great stability, deep-sea extremozymes have numerous potential applications in a wide range of industries, such as the agricultural, food, chemical, pharmaceutical and biotechnological sectors. This enormous economic potential combined with recent advances in sampling and molecular and omics technologies has led to the emergence of research regarding deep-sea extremozymes and their primary applications in recent decades. In the present review, we introduced recent advances in research regarding deep-sea extremophiles and the enzymes they produce and discussed their potential industrial applications, with special emphasis on thermophilic, psychrophilic, halophilic and piezophilic enzymes.

## 1. Deep-Sea Extremophilic Microorganisms: A Novel Source of Extremozymes

Nearly three-quarters of the Earth’s surface area is covered by ocean, the average depth of which is 3800 m, implying that the vast majority of our planet comprises deep-sea environments. The deep sea is one of the most mysterious and unexplored environments on the Earth, and it supports diverse microbial communities that play important roles in biogeochemical cycles [1]. The deep sea is also recognised as an extreme environment, as it is characterised by the absence of sunlight and the presence of predominantly low temperatures and high hydrostatic pressures, and these environmental conditions become even more challenging in particular habitats, such as deep-sea hydrothermal vents with their extremely high temperatures of >400 °C, deep hypersaline anoxic basins (DHABs) with their extremely high salinities and abysses of up to 11 km depth with their extremely high pressures. 

Deep-sea extremophiles are living organisms that can survive and proliferate in deep-sea environments that have extreme physical (pressure and temperature) and geochemical (pH, salinity and redox potential) conditions that are lethal to other organisms. The majority of deep-sea extremophiles belong to the prokaryotes, which are microorganisms in the domains of Archaea and Bacteria [2,3]. These extremophilic microorganisms are functionally diverse and widely distributed in taxonomy [4], and they are classified into thermophiles (55 °C to 121 °C), psychrophiles (−2 °C to 20 °C), halophiles (2–5 M NaCl or KCl), piezophiles (>500 atmospheres), alkalophiles (pH > 8), acidophiles (pH < 4) and metalophiles (high concentrations of metals, e.g., copper, zinc, cadmium and arsenic) according to the extreme environments in which they grow and the extreme conditions they can tolerate. Many deep-sea extremophiles tolerate more than one extreme condition, and thus are polyextremophiles [5]. These extreme conditions are generally harmful to the majority of organisms, but extremophilic microorganisms are able to survive and thrive in them due to their highly flexible metabolisms and the unique structural characteristics of their biomacromolecules [6,7]. 

In the past few decades, deep-sea extremophilic microorganisms have attracted the attention of researchers searching for novel bioactive substances such as enzymes that can be used in the major sectors of industry worldwide [8]. The diverse temperatures, salinities, pHs and pressures that are provided by nature in extreme deep-sea environments can be utilised to search for novel and potentially robust enzymes that are more suitable for industrial applications [9], and it has been found that the extremozymes that are produced by deep-sea extremophilic microorganisms have a wide variety of industrial applications due to their high activities and great stabilities under extreme conditions. Indeed, the stability and enzymatic activities of extremozymes make them valuable alternatives to ordinary biotechnological processes, bestowing them with considerable economic potential in the agricultural, feed, food, beverage, pharmaceutical, detergent, leather, textile, pulp and biomining industries [10]. 

Although a lot of enzymes have been identified to date worldwide, the majority of which have been evaluated for industrial applications, the enzyme market remains inadequate in meeting industrial demands [11] largely due to many of the enzymes that are presently available being unable to tolerate industrial conditions [12]. The industrial process demands biocatalysts that can resist a range of harsh conditions, including temperature, pH, salinity and pressure, while exhibiting high conversion rates and reproducibilities [13]. Furthermore, it is important that the enzymes that are used in technologies are compatible with ecological processes [14]. While only a few extremozymes are presently being produced and used at the industrial level, the development of novel industrial processes based on these enzymes is being promoted by advances in deep-sea extremophile and extremozyme research, the growing demand for novel biocatalysts in industries, breakthroughs in deep-sea sampling techniques and the rapid development of new molecular and omics technologies, such as metagenomics, proteomics, protein engineering, gene-directed evolution and synthetic biology [15]. Thus, the discovery of enzymes with novel enzymatic activities and improved stability remains a priority in enzyme research [10]. 

## 2. Strategies for Discovering Extremozymes in Deep-Sea Environments

The classic method that is used to discover novel extremozymes from deep-sea microorganisms is the cultivation of microorganisms followed by screening for the desired enzymes. However, while numerous extremozymes with promising properties for industrial applications have been isolated from deep-sea environments using this method, approximately 99.9% of those environmental microorganisms cannot be cultivated using traditional laboratory techniques [16], meaning that the discovery of many useful extremozymes would not be possible using this method alone. Metagenomic technologies have been developed to bypass the requirement for the isolation or cultivation of microorganisms, and they could prove to be a powerful tool for discovering novel genes and enzymes directly from uncultured microorganisms [17]; indeed, metagenomes have been successfully employed to search for extremozymes from deep-sea environments, overcoming the bottlenecks associated with the uncultivability of extremophiles [18].

Metagenomic analyses are based on the direct isolation of genomic DNA from environmental samples, and they are either sequence based (i.e., putative enzymes are obtained based on their conserved sequences) or function based (i.e., functional enzymes are obtained based on the expressed features such as a specific enzyme activity) [19]. In the sequence-based approach, the colony hybridisation technique is used for screening metagenomic clones using an oligonucleotide primer or probes for the target gene, and the desired gene may also be amplified by polymerase chain reaction (PCR) using specific or degenerate primers and subsequently cloned into suitable expression vectors. Besides, the desired gene sequences can sometimes be directly retrived from metagenomics data after proper bioinformatic annotations, and then be synthesized de novo and codon-optimized if required. This sequence-based technique leads to the discovery of novel sequences that are similar to existing known sequences, and this provides the possibility of finding enzymes efficiently [20]. However, the ability to identify specific enzymes using this method depends on existing bioinformatic analyses, thus many novel or unknown activities can be overlooked [21]. 

The common function-based metagenomic strategies include enzyme activity-based screening performed in culture plates; for example, the use of the starch-iodine staining test for detecting amylase activity. Functional metagenomics has some advantages over the sequence-based approach because the identification of genes according to their functions rather than their sequences eliminates the possibility of incorrect annotations or obtaining similar sequences of gene products with different or multiple functions. Furthermore, functional screening is more suitable for identifying novel genes encoding novel enzymes because it does not rely on gene sequence information [22]. The primary disadvantage of this screening method is that gene expression may fail due to difficulties in promoter recognition, low translation efficiency, lack of specific cofactors in certain expression hosts, protein misfolding and post-translational modification defects of the desired proteins. However, all these issues can be solved using vectors with a wide host range that enables expression in a variety of hosts, vectors that are adapted to a large insert size and Rosetta strains of *Escherichia coli*, which contains transfer ribonucleic acid (tRNA) for rare amino acid codons [17,23]. Consequently, the function-based metagenomic approach is now the most frequently used technique for screening for novel extremozymes from the deep sea [24,25,26,27].

The application of enzymes in industrial processes sometimes fails due to the presence of undesirable properties and a lack of stability and robustness [28]. However, molecular approaches can be used to engineer natural proteins and develop more effective extremozymes with enhanced stabilities and activities for industrial purposes. The enhancement of the stability of enzymes can prove to be very beneficial because it would enable them to maintain high activity for prolonged periods of time under challenging physicochemical conditions, which would be a useful characteristic for numerous industrial processes. One method that can be used to stabilise proteins is protein engineering [29,30], which has become a powerful approach for altering or improving enzymatic characteristics in the past two decades. Protein engineering is divided into the following two methods: (1) directed evolution, where a random mutagenesis is applied to a protein [31]; and (2) rational protein design, where knowledge of the structure and function of the protein is exploited to modify its characteristics [32]. Both these methods have been successfully applied to increase the activity, selectivity and thermostability of proteins. 

## 3. Properties and Applications of Extremozymes Isolated from Deep-Sea Extremophilic Microorganisms

Hundreds of industrial processes and products benefit from the use of enzymes that have been isolated from microorganisms. However, the majority of the enzymes that are presently on the market are produced using mesophilic enzymes, which are often inhibited under the extreme conditions of several industrial processes [10]. In addition, the stability of the biocatalyst is important for reducing costs because enzymes that are sufficiently stable to withstand the industrial conditions can be used for repeated cycles of the biocatalytic process, and hence aid in reducing expenditures. Thus, the exploration of deep-sea extremophilic microorganisms provides an opportunity for obtaining extremozymes that are stable under a variety of different conditions, which may be attractive in industrial processes. Furthermore, enzymes that catalyse reactions under non-physiological conditions and/or with non-natural substrates can also be found in deep-sea environments [33]. Consequently, deep-sea extremozymes have received increasing attention for their applications in various industrial processes owing to their adaptability to harsh physical and chemical conditions [11]. 

### 3.1. Deep-Sea Thermophilic Enzymes

Deep-sea thermophiles have been one of the most studied groups of extremophiles over the past four decades [34,35]. These microorganisms are able to grow at high temperatures of 41 °C–120 °C [36,37], therefore, they produce extremozymes with high-temperature resistance. These thermophilic enzymes use a variety of mechanisms to tolerate extreme temperatures, possessing electrostatic interactions and physical properties that allow them to maintain their activity. In general, thermophilic enzymes have similar three-dimensional structures to their mesophilic counterparts but have many more charged residues on their surfaces and different amino acid contents. In addition, thermophilic enzymes usually have shorter loops, thereby inhibiting nonspecific interactions that are induced by their increased flexibility at high temperatures [38,39]. Thermophilic enzymes also have increased number of bisulphide bonds formed between two cysteine residues, which enhances their structural rigidity and, thus, resistance to unfolding at high temperatures [40,41]. 

To date, thermophilic enzymes have attracted the most attention among the various types of extremozymes because enzymes that are adapted to higher temperatures have several important advantages to industrial processes. High temperatures not only significantly increase the solubility of many reagents, particularly polymeric substrates, but also reduce the risk of contamination, which would result in unfavourable complications. Moreover, high temperatures also promote faster reactions, maintain a low viscosity and increase solvent miscibility [42]. Thermophilic enzymes are usually capable of accepting proteolysis and extreme conditions, such as the presence of organic solvents, denaturing agents and high salinity, making them attractive in the biorefinery, paper and bleaching, and first- and second-generation biofuel industries [43]. A large number of enzymes from deep-sea thermophilic microorganisms have been characterised to date (Table 1) [35], and thermophilic proteases, lipases and polymer-degrading enzymes, in particular, have found their way into industrial applications [11]. 

The diversity of deep-sea thermophiles makes them valuable in the search for novel thermostable proteolytic enzymes [56,57], which are attractive for use in the detergent, food and feed industries. Jin et al. [44] reported the purification and characterisation of a thermostable and alkali-stable keratinase Ker02562 from *Bacillus* sp. JM7, which was isolated from the deep sea. This enzyme was shown to be stable at 50 °C and in extreme alkaline environments (pH 10–13) and so may have significant applications in the detergent industry, as the enzymes that are used in detergent additives need to be able to withstand temperatures of 40–60 °C and an alkaline pH (pH 9.0–11.0) [58]. Other thermophilic proteases with important industrial applications include thermolysin, which is used in the synthesis of dipeptides, DNA-processing enzymes and pretaq protease, which is used to clean up DNA before PCR amplification [59]. For example, the proline dipeptidase named prolidase, which was identified from the archaeon hyperthermophile *Pyrococcus furiosus* isolated from deep-sea vents and volcanic marine mud in Italy and specifically cleaves dipeptides with proline at the C-terminus and a nonpolar residue (Met, Phe, Val, Leu, Ala) at the amino terminus, is by far the most thermostable example of a prolidase known to date, with a temperature optimum above 100 °C and no loss of activity after 12 h at this temperature [45].

Industrial and biotechnological processes also require thermostable lipases for use in processes such as grease esterification, hydrolysis, transesterification, interesterification and organic biosynthesis. Zhu et al. [46] cloned and characterised a thermostable lipase from the deep-sea hydrothermal field thermophile *Geobacillus* sp. EPT9 and found that the recombinant lipase was optimally active at 55 °C and pH 8.5 and exhibited good thermostability, retaining 44% residual activity after incubation at 80 °C for 1 h. A thermostable monoacylglycerol lipase (GMGL) has also been identified from the thermophilic bacterium *Geobacillus* sp. 12AMOR1, which was isolated from a deep-sea hydrothermal vent site in the Arctic [47]. GMGL is active on monoacylglycerol substrate but not diacylglycerol or triacylglycerol, and recombinant GMGL shows the highest hydrolysis activity at 60 °C and pH 8.0 and has a half-life of 60 min at 70 °C. These thermostable lipases have considerable potential for applications in the food, detergent, cosmetics, perfumery, pharmaceutical, pulp and paper, and chemical industries [60]. 

Deep-sea thermostable polymer-degrading enzymes, such as agarases, amylases and cellulases, are another group of industrially important biocatalysts that have received much interest. Agarases catalyse agar hydrolysis and have been successfully utilised to produce agar-oligosaccharides, which possess a variety of biological and physiological functions that are beneficial to human health and thus have potential applications in the food and nutraceutical industries [14,61,62,63]. Since the gelling temperature of agar is approximately 40 °C, thermostable agarases are required for the efficient recovery of DNA from agar gel and are also advantageous for the industrial production of oligosaccharides from agar. Hou et al. reported the expression and characterisation of a novel thermostable and pH-stable β-agarase AgaP4383 from the deep-sea bacterium *Flammeovirga pacifica* WPAGA1 [49]. Phenotypic and genomic analyses revealed that *F. pacifica* WPAGA1 is capable of degrading and metabolising complex polysaccharides and can grow on the red alga *Gracilaria lemaneiformis* as a sole carbon source [64,65], and that AgaP4383 exhibits endolytic activity on agar degradation, producing neoagarotetraose and neoagarohexaose as the final products. AgaP4383 also exhibits good thermostability, with no loss of activity after incubation at 50 °C for 10 h [63]. Recently, a novel thermostable and pH-stable β-agarase Aga4436 was reported from another deep-sea bacterium in the genus *Flammeovirga*, *Flammeovirga* Sp. OC4, which also shows high activity and stability at high temperatures [48]. These favourable properties of AgaP4383 and Aga4436 could make them attractive for use in the food and biotechnology industries.

Thermostable amylolytic enzymes are one of the most interesting groups of enzymes for industrial processes, as they are important for the hydrolysis of starch at high temperatures, promoting the reactions and reducing the risk of contamination [66]. Several thermostable amylases have been reported from deep-sea microorganisms [51,52,67], some of which have been developed into products. For example, Fuelzyme^®^, a product from Verenium Corporation (San Diego, CA, USA), utilises an alpha-amylase from the thermophile *Thermococcus* sp., which was isolated from a deep-sea hydrothermal vent. Fuelzyme^®^ operates in extremely high temperatures (>110 °C) and at an acidic pH (4.0–6.5), making it suitable for mash liquefaction during ethanol production, releasing dextrins and oligosaccharides with lower molecular weights and better solubilities [67]. However, Fuelzyme^®^ and Spezyme^®^ (DuPont-Genencor Science, Wilmington, DE, USA) are only presently used in the production of biofuel. It has been proposed that the combined use of these commercially available amylases and other *Bacillus* amylases will increase the efficiency of industrial starch processing and will be suitable for downstream applications [68]. 

Thermophilic enzymes are also widely used in industrial lignocellulolytic processes, with thermostable cellulases having applications in the food, animal feed, textile, and pulp and paper industries [7]. Functional screening of fosmid expression libraries derived from deep-sea hydrothermal vents identified an extreme cellulase that was active and thermostable at 92 °C. This enzyme showed endolytic activities against a variety of linear 1,4-β-glucans, such as phosphoric acid swollen cellulose, carboxymethyl cellulose, lichenan and β-glucan. Other industrial important thermostable polymer-degrading enzymes have also been identified and characterised from deep-sea environments, such as amylopullulanases, arylsulphatases and xylanases [53,54,55]. 

### 3.2. Deep-Sea Psychrophilic Enzymes

The deep sea is primarily a cold environment, and the majority of the water is present at 5 °C. Consequently, this biome harbours an abundance of cold-adapted psychrophiles, which have a restricted range of temperature for growth, and are extremophiles that are adapted to moderate or extreme cold and have been shown to achieve active metabolism at −25 °C and conduct DNA synthesis at −20 °C [69]. Psychrophiles are divided into the following two types according to their growth temperatures: eurypsychrophiles (formerly psychrotolerant microorganisms), which comprise the majority of isolates from the deep sea and have a broad temperature range and tolerate warmer environments, and stenopsychrophiles (formerly true psychrophiles), which cannot grow at temperatures above 20 °C [70]. Psychrophiles have developed several mechanisms that allow them to thrive in icy environments, including the production of cold-induced cold-shock proteins and RNA chaperones, enhanced tRNA flexibility, enhanced membrane fluidity for maintaining the semi-fluid state of the membranes, and the production of cold-active secondary metabolites, enzymes, pigments and antifreeze proteins [71,72]. The most common adaptive characteristic of psychrophilic enzymes is their high reaction rate at low temperatures, which is generally achieved by their flexible structures and low stabilities [73]. From a structural perspective, psychrophilic proteins have a higher content of α-helix than β-sheets, which is recognised as an essential feature for maintaining flexibility even at low temperatures [7]. Because cold-active enzymes maintain a high catalytic rate at low temperatures through the augmentation of the solvent connection and structural flexibility [73,74], they are capable of binding more tightly to the solvent, in a way similar to that of salt-adapted enzymes [75].

Enzymes that are adapted to low temperatures possess several features that are favourable for industrial applications [76], and have been used in industries as diverse as food processing, molecular biology and fine chemical synthesis [77,78]. Cold-active enzymes bring potential benefits to the food and feed industries, where it is crucial to avoid spoilage as this may result in a change in nutritional value and flavour of the original thermosensitive substrates and products [77,79]. Cold-adapted enzymes are also useful for molecular biology because of the need to use enzymes in sequential reactions and to inactivate enzymes once they have accomplished their functions. To this end, heat-labile enzymes have great potential, as heat inactivation can be performed at temperatures that do not cause the melting of double-stranded DNA (dsDNA), eliminating the need for additional chemical extraction steps [77]. The detergent, biofuel production and pulp and paper industries are also interested in cold-active hydrolases, such as proteases, lipases, amylases and cellulases, as these enzymes can provide economic benefits by reducing energy consumption and production costs [80,81].

Deep-sea psychrophiles are a promising source of industrially important cold-active enzymes (Table 2). Cold-active cellulose-degrading enzymes, such as glucosidases, are useful in the textile, beverage and biofuel industries. The first cold-active and alkali-stable β-glucosidase was isolated from the deep-sea bacterium *Martelella mediterranea* and showed favourable characteristics, being able to retain more than 50% of its maximum enzymatic activity at 4 °C and 80% of its maximum enzymatic activity at pH 11 for 11 h [82]. The deep-sea-sediment–dwelling bacterium *Exiguobacterium oxidotolerans* also produces a cold-active β-glycosidase, which maintains 61% of its maximum activity at 10 °C and in a pH range of 6.6–9.0 [83]. Several cold-adapted xylanases that have been isolated from the deep sea, such as from *Zunongwangia profunda* [84] and *Flammeovirga pacifica* [85], have potential uses in the food industry, such as as additives to wheat flour to improve dough handling and the quality of the baked products. Numerous cold-adapted amylases have also been isolated from deep-sea microorganisms over the past few years that have potential applications in the detergent, textile and food industries [86,87,88]; for example, Jiang et al. [89] reported a cold-adapted alpha-amylase from the deep-sea bacterium *Bacillus* sp. dsh19-1, which shows maximum activity at 20 °C. Cold-active lipases and esterases are important catalysts in the chemical, pharmaceutical, cosmetic, food, laundry detergent and environmental remediation industries and can be isolated from deep-sea microorganisms and metagenomic libraries derived from deep-sea samples [77]. For example, Chen reported a novel psychrophilic esterase Est11 from the deep-sea bacterium *Psychrobacter pacificensis*, which is highly active and stable at 10 °C and 5 M NaCl [90]. Furthermore, incubation with ethanol, isopropanol, propanediol, dimethyl sulphoxide (DMSO), acetonitrile and glycerol were shown to have remarkable positive effects on Est11 activity, indicating that this cold-active, halo-tolerant and organic solvent-resistant esterase may be useful in harsh industrial processes [90]. 

### 3.3. Deep-Sea Halophilic Enzymes

Halophiles are capable of thriving in high salt concentrations, which can be found in DHABs or deep-sea hypersaline anoxic lakes. These extreme habitats have been discovered on the sea floor in different oceanic regions, such as the Red Sea, the eastern Mediterranean Sea and the Gulf of Mexico. In a DHAB, the dissolved evaporitic deposits are trapped in the sea floor sediments, forming very stable brine and a sharply stratified chemocline in the water column. The brines that are enclosed in these basins are characterised by hypersalinity (5–10 times the concentration of sea water), a high pressure (approximately 35 MPa), a lack of oxygen and highly reducing conditions, and an absence of light, making them one of the most extreme environments on the Earth, which has allowed these habitats to remain isolated for thousands of years [7,96]. Since the discovery of the first Mediterranean DHAB termed ‘Tyro’ in 1983, six more DHABs have been discovered termed ‘l’Atalante’, ‘Bannock’, ‘Discovery’, ‘Medee’, ‘Thetis’ and ‘Urania’ [97], all of which are sources of anaerobic halophilic microorganisms. 

Halophilic microorganisms can be classified into the following three categories according to the optimal salt concentration for growth: (i) slight halophiles, which are capable of developing at 200–500 mM NaCl; (ii) moderate halophiles, which can develop at 500–2500 mM NaCl; and (iii) extreme halophiles, which can develop at 2500–5200 mM NaCl [98]. Certain halophiles are also thermostable and tolerant to a wide range of pHs, and halophiles have high metabolic diversity, comprising anoxic phototrophic, fermenter, aerobic heterotrophic, sulphate reducer, denitrifying and methanogenic organisms [99].

Halophiles have developed different adaptive strategies to survive the osmotic pressures that are induced by the high NaCl concentrations in the environments they inhabit. Some extremely halophilic bacteria use a type of ‘salt-in’ strategy to balance the osmotic pressure of the environment, whereby they accumulate inorganic ions (K^+^, Na^+^, Cl^−^) in the cytoplasm [100]. However, moderate halophiles have distinct adaptations that allow them to biosynthesize and/or accumulate large amounts of specific organic osmolytes in the cytoplasm. These accumulated osmolytes act as osmoprotectants and help to maintain osmotic balance and low salt concentrations in the cytoplasm without interfering with the normal cellular metabolism [101]. Halophiles also produce enzymes that are active and stable in the presence of salts, with the enzymes employing different adaptation mechanisms and exhibiting very high stability at low water activity as well as in the presence of organic solvents and high salt concentrations [35,102]. Structural analyses have revealed that the major differences between non-halophilic and halophilic proteins occur on the surfaces of the molecules. Halophilic enzymes contain a greater percentage of certain amino acid residues, such as serine and threonine, a higher proportion of aspartic and glutamic acids, a lower percentage of lysine, and a higher occurrence of amino acids with low hydrophobic characters than non-halophilic enzymes, which allow a higher number of salt bridges to be created and cooperation with electrostatic interactions [103]. The stability of these enzymes relies on the negative charge of the acidic amino acids on the protein surface, the hydration of the protein surface due to the carboxylic groups that are present in aspartic and glutamic acids, and the occurrence of hydrophobic groups in the presence of high salt concentrations. In addition, the negative surface charges are believed to be important for the solvation of halophilic proteins and the prevention of denaturation, aggregation and precipitation [101,104]. Deep-sea halophilic enzymes provide great opportunities for the food, detergents, textile, bioremediation and biosynthetic industries [102], with their industrial potential lying in their activity and stability not only at high salt concentrations but also in the presence of organic solvents [105,106]. Moreover, several deep-sea halophilic enzymes are also active and stable at high or low temperatures [89,90]. These unique properties make deep-sea halophilic enzymes attractive wherever enzymatic conversion needs to occur under challenging physical and chemical conditions, such as at extreme salt concentrations and temperatures and in the presence of organic solvents. 

Numerous halophilic enzymes isolated from the deep sea have been cloned and characterised to date (Table 3), with examples of industrially important halophilic enzymes including polysaccharide-hydrolysing enzymes, such as amylases and xylanases [10,102,107]. For instance, two cold-adapted and salt-tolerant alpha-amylases have been reported from the deep-sea bacteria *Bacillus* sp. dsh19-1 and *Zunongwangia profunda*, which are among the very few known alpha-amylases that can tolerate both cold and saline conditions [88,89]. The high activities and stabilities of these halophilic amylases in harsh conditions make them desirable for industrial applications, particularly for the treatment of waste water containing high salt concentrations and starch residues. In addition, a novel psychrophilic and halophilic β-1,3-xylanase (Xyl512) was recently characterised from the deep-sea bacterium *Flammeovirga pacifica* strain WPAGA1, and it was found that a high-saline concentration (1.5 M NaCl) could alter the optimum temperature and pH of Xyl512, as well as significantly improve its overall activity by two-fold compared with an absence of NaCl, which would meet the food industry’s demands for low temperatures and high concentrations of salt [85]. Some deep-sea halophilic enzymes are lipolytic, such as esterases, which have particularly great potential in the production of biodiesel, polyunsaturated fatty acids and food [108,109]. Five esterase genes have been identified from a metagenome expression library derived from the DHAB Urania, some of which were highly active in high-salinity conditions and were able to function in polar solvents, making them suitable for use in the chemical, pharmaceutical and biofuel industries [110]. In addition, research regarding deep-sea extremophiles has identified and characterised other industrially important halophilic enzymes, such as proteases, mercuric reductase and the first reported DNA polymerase to exhibit halophilic and thermophilic features [111,112,113].

### 3.4. Deep-Sea Piezophilic Enzymes

The hydrostatic pressure can reach 70–110 MPa in the deepest parts of the oceans, making these environments highly challenging. These deep-sea habitats host a group of extremophiles known as piezophiles, which survive and thrive under conditions of extremely high hydrostatic pressure [115,116]. Piezophiles can be classified into the following two categories based on their pressure requirements for growth: 1) piezophilic microorganisms, which exhibit optimal growth at pressures above atmospheric pressure; and 2) piezotolerant microorganisms, which can grow at atmospheric pressure and high pressures but do not require high pressures for optimal growth [117]. High pressure plays a selective role on living organisms by affecting cellular structures and processes, such as cell motility and division. Consequently, the ability to live under extreme pressures requires substantial physiological adaptations that involve modifications to gene regulation and the cellular structure. The adaptative mechanisms of piezophiles have not yet been fully clarified but are known to involve a reduction in cell division, the production of compatible osmolytes and polyunsaturated fatty acids, a switch in the flexibility state, and the formation of multimeric and antioxidant proteins [118,119,120,121]. Lauro et al. have also described the occurrence of extended helices in the 16S ribosomal RNA (rRNA) genes for adaptation to high pressures [122].

So far, very little research has been conducted on deep-sea piezophilic enzymes [117,123], and many more experiments and computational studies on different enzymes from a variety of piezophiles are required to advance our understanding [120]. However, piezophilic proteins have shown high efficiency in several industrial processes [5], with particular applications for food production, where high pressures are employed for the processing and sterilisation of food materials [124]. For example, piezophilic α-amylase has been shown to produce trisaccharide instead of maltobiose and tetrasaccharide from maltooligosaccharide at high pressures with little energy, which is a useful reaction in the food processing industry [35,117]. In addition, a peptidase from *Pyrococcus horikoshii* demonstrates stability at high pressures and thus may be useful for food processing [35,125]. Deep-sea piezophiles often produce polyunsaturated fatty acids, such as omega-3 polyunsaturated fatty acids, to stabilise the cell membrane under high pressure. This increase in unsaturated fatty acids creates highly disordered phospholipid bilayers, which renders the membrane resistant against high pressure [116]. Thus, the lipid biochemistry of piezophilic microorganisms is a very interesting topic, and the enzymes that participate in these metabolic pathways under high pressures may have great potential for industrial applications [126]. Piezophilic endonucleases may also have potential in the biotechnology industry. For example, the ‘star activity’ that is exhibited by *Eco*RI and other restriction endonucleases under high-osmotic-pressure conditions, whereby they lose some specificity to their recognition sequences, can be reversed by piezophilic endonucleases operating under a hydrostatic pressure of 50–75 Mpa [117,127].

## 4. Conclusions and Prospects

Deep-sea extremophiles are emerging as an important source of novel industrially robust extremozymes. The biodiversity of deep-sea extremophiles and the evolutionary adaptations of their derived extremozymes to the harsh conditions that are found in deep-sea ecosystems has facilitated the selection of more robust biocatalysts, which have special properties that are not found in any other prokaryotes. These extremozymes will be beneficial for novel biocatalytic processes, allowing them to be more efficient, specific, accurate and environmentally friendly. Due to their special features and enormous potential in industrial applications, the number of studies on deep-sea extremophiles and their extremozymes has greatly increased over the last two decades. However, there are still only a few extremophiles available and only a minor fraction of the deep-sea extremophiles has been exploited for extremozymes to date. This limited exploitation of extremozymes is largely due to the special nutritional requirements and challenging growth conditions of deep-sea extremophiles, which make their isolation and maintenance difficult. Fortunately, however, the rapidly increasing number of extremophilic genomes and metagenomes that can now be easily obtained by next-generation sequencing technologies offers an ever-expanding resource for the identification of new extremozymes from non-cultivable deep-sea extremophiles. In addition, although enzyme engineering techniques have been established, the enzyme optimisation process is still a limiting factor for the development of new extremozyme-inspired industrial processes. Thus, the simultaneous development of protein engineering technologies will assist the further modification and improvement of biocatalytic features, which will increase the application of deep-sea extremophiles in industry. Moreover, important advances in our knowledge of the genetics, physiology, metabolism and enzymology of deep-sea extremophiles are expected, which will enable us to better understand the applications of their biocatalysts. Thus, the advancement of modern molecular techniques and deep-sea sampling approaches in the future will allow deep-sea extremozymes to have significant impacts on a wide range of industries. 

## Figures and Tables

**Table 1:** Representative thermophilic enzymes from deep-sea microorganisms.

Source	Habitat	Enzyme	Thermostability	References
*Bacillus* sp. JM7	deep-sea water	keratinase	50 °C (70%, 1 h)	[44]
*Pyrococcus furiosus*	deep-sea vents	prolidase	100 °C (100%, 12 h)	[45]
*Geobacillus* sp. EPT9	deep-sea vents	lipase	80 °C (44%, 1 h)	[46]
*Geobacillus* sp. 12AMOR1	deep-sea vents	monoacylglycerol lipase	70 °C (half-life 1 h)	[47]
*Flammeovirga* Sp. OC4	deep-sea water	β-Agarase	50 °C (35%, 144 h)	[48]
*Flammeovirga pacifica*	deep-sea water	β-Agarase	50 °C (100%, 10 h)	[49]
*Microbulbifer* strain JAMB-A7	deep-sea sediment	β-Agarase	50 °C (half-life 502 min)	[50]
*Flammeovirga pacifica*	deep-sea water	α-amylase	60 °C (81%, 20 min)	[51]
*Geobacillus* sp. 4j	deep-sea sediment	α-amylase	80 °C (half-life 4.25 h)	[52]
Fosmid library	deep-sea vents	cellulase	92 °C (half-life 2 h)	[24]
*Flammeovirga pacifica*	deep-sea water	arylsulfatase	50 °C (70%, 12 h)	[53]
*Staphylothermus marinus*	deep-sea vents	amylopullulanase	100 °C (half-life 50 min)	[54]
*Geobacillus sp. MT-1*	deep-sea vents	xylanase	65 °C (half-life 50 min)	[55]

**Table 2:** Representative psychrophilic enzymes from deep-sea microorganisms.

Source	Habitat	Enzyme	Activities at Low Temperatures	References
*Martelella mediterranea*	deep-sea water	β-glucosidase	50% at 4 °C	[82]
*Exiguobacterium oxidotolerans*	deep-sea sediment	β-glycosidase	61% at 10 °C	[83]
*Zunongwangia profunda*	deep-sea sediment	xylanase	38% at 5 °C	[84]
*Flammeovirga pacifica*	deep-sea water	xylanase	50–70% at 10 °C	[85]
*Luteimonas abyssi*	deep-sea water	α-amylase	36% at 10 °C	[87]
*Zunongwangia profunda*	deep-sea sediment	α-amylase	39% at 10 °C	[88]
*Pseudomonas* strain	deep-sea sediment	α-amylase	50% at 5 °C	[91]
*Wangia* sp. C52	deep-sea sediment	α-amylase	50% at 25 °C	[92]
*Bacillus* sp. dsh19-1	deep-sea sediment	α-amylase	35.7% at 4 °C	[89]
*Psychrobacter pacificensis*	deep-sea water	esterase	70% at 10 °C	[90]
Metagenomic libraries	deep-sea sediment	esterase	100% at 10 °C	[25]
Metagenomic libraries	deep-sea sediment	esterase	38% at 15 °C	[26]
Metagenomic libraries	deep-sea sediment	lipase	most active below 30 °C	[93]
*Pseudoaltermonas* sp. SM9913	deep-sea sediment	serine protease	60% at 20 °C	[94]
*Planococcus* sp. M7	deep-sea sediment	protease	45% at 10 °C	[95]

**Table 3:** Representative halophilic enzymes from deep-sea microorganisms.

Source	Habitat	Enzyme	Activities at High Saline Concentrations	References
*Zunongwangia profunda*	deep-sea sediment	α-amylase	93% activity at 4 M NaCl	[88]
*Bacillus* sp. dsh19-1	deep-sea sediment	α-amylase	60.5% activity at 5 M NaCl	[89]
*Zunongwangia profunda*	deep-sea sediment	xylanase	near 100% activity at 5 M NaCl	[84]
*Flammeovirga pacifica*	deep-sea water	xylanase	maximum at 1.5 M NaCl	[85]
*Emericellopsis* sp. TS11	deep-sea sponge	xylanase	maximum at 2 M NaCl	[114]
Metagenomic libraries	deep-sea brine	esterase	maximum at 3–4 M NaCl	[110]
Fosmid library	deep-sea sediment	esterase	maximum at 3.5 M NaCl	[27]
*Psychrobacter pacificensis*	deep-sea water	esterase	maximum at 5 M NaCl	[90]
*Pseudoalteromonas* spp.	deep-sea sediment	protease	maximum at 2 M NaCl	[111]
Metagenomic libraries	deep-sea brine	mercuric reductase	maximum at 4 M NaCl	[112]
candidate division MSBL1 archaeon SCGC-AAA261G05	deep-sea brine	DNA polymerase	maximum at 0.5 M NaCl	[113]
